# Pulmonary manifestations of IgG4‑related disease in a South African patient

**DOI:** 10.7196/AJTCCM.2021.v27i1.130

**Published:** 2021-03-09

**Authors:** J D Cilliers, S C Eindhoven, E Louw, C F N Koegelenberg, E Irusen, C Bruce-Brand, B W Allwood

**Affiliations:** 1 Division of Pulmonology, Department of Medicine, Faculty of Medicine and Health Sciences, Stellenbosch University and Tygerberg Hospital, Cape Town, South Africa; 2 Department of Respiratory Medicine, Franciscus Gasthuis and Vlietland, Rotterdam, The Netherlands; 3 Division of Anatomical Pathology, Department of Pathology, Faculty of Medicine and Health Sciences, Stellenbosch University, Cape Town, South Africa; 4 National Health Laboratory Service, Tygerberg Hospital, Cape Town, South Africa

**Keywords:** IgG4 related disease, IgG4 pulmonary involvement, haemoptysis

## Abstract

Immunoglobin 4-related disease (IgG4-RD) is an auto-immune, multisystem inflammatory disorder characterised by storiform fibrosis, lymphoplasmacytic infiltration and obliterative phlebitis on histology. Its pathophysiology is not well understood, but is thought to occur due to complex interactions between T helper 2 cells, their cytokines, chemokines, and B lymphocytes that become dysregulated and produce dysfunctional immunoglobulins. Here, we present a case report of a 54-year-old man who was initially suspected of having lung cancer on imaging, but was ultimately diagnosed with IgG4-RD on histological analysis of a pneumonectomy specimen. Treatment with glucocorticoids can establish disease remission, with a small proportion of patients relapsing, if the diagnosis is made before significant fibrosis occurs.

## Case

**Fig. 1:**
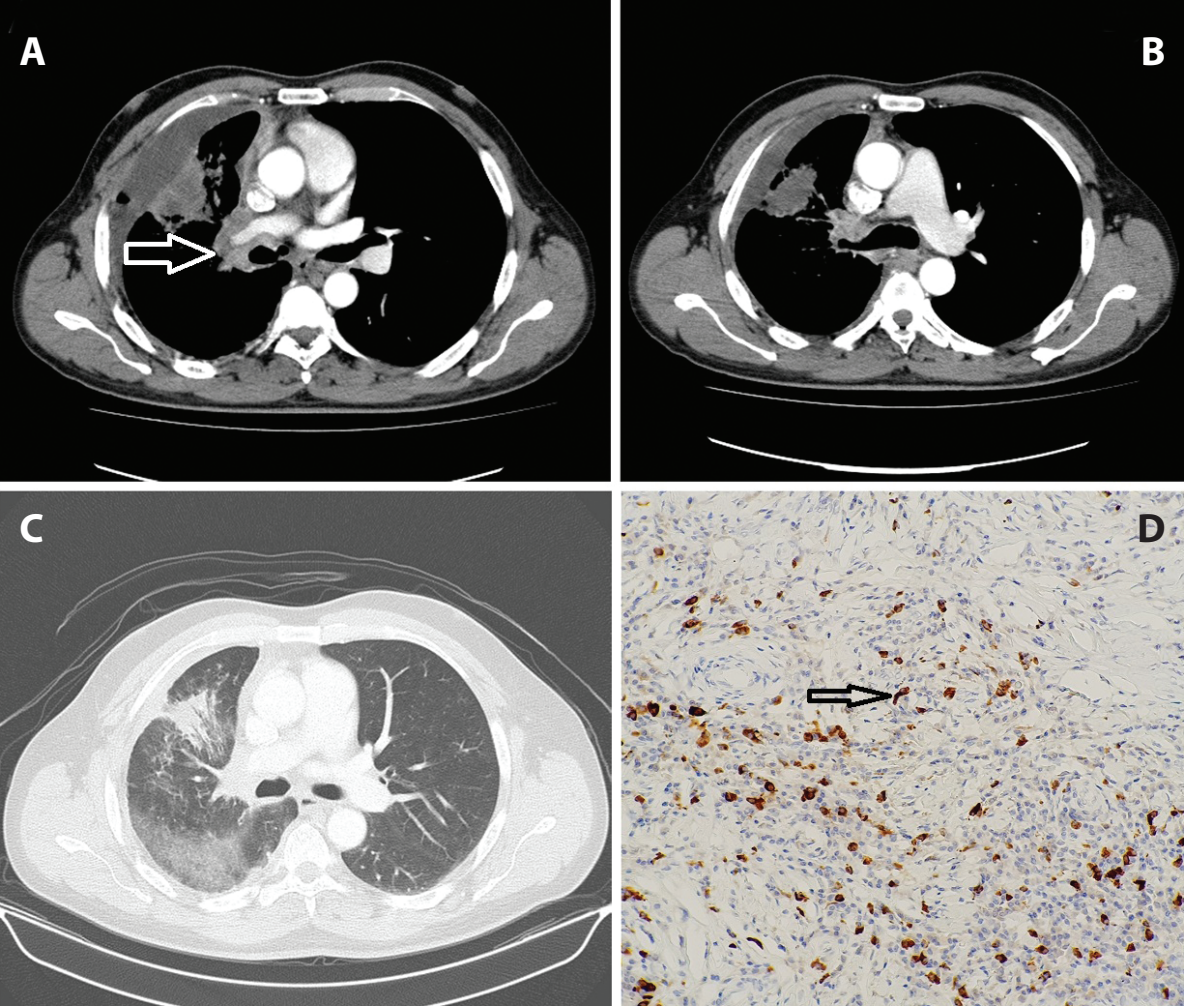
A (arrow) - Axial computed tomography images showing right hilar nodal complex. B - collapse consolidation of basal segment of the right upper lobe. C - and right-sided pleural effusion. D (arrow) - Histology of resected lung showing storiform fibrosis and IgG4-positive plasma cells

A 54-year-old man with a background of well-controlled type 2 diabetes mellitus, hypertension, dyslipidaemia and benign prostatic hypertrophy was referred to Tygerberg Hospital with a non-productive cough, pleural effusion and abnormal chest X-ray. He was a smoker with a 25-pack a year smoking history and a presumptive diagnosis of stage IV lung carcinoma was considered based on computed tomography (CT) findings (Fig. 1A - C).

He reported moderate dyspnoea (modified Medical Research Council grade 2) and pulmonary function tests (PFTs) demonstrated a forced expiratory volume in 1 second (FEV_1_ ) of 3.02 L (91% predicted), a forced vital capacity (FVC) of 4.48 L (110% predicted) and FEV_1_ /FVC of 67%. His diffusing capacity for carbon monoxide was 4.28 L (98.8% of predicted) and total lung capacity (TLC) was 5.76 L (88% predicted), with a residual volume:TLC ratio of 0.41. He did not report any other systemic complaints and his problems appeared to be isolated to the lungs. Notably, he had no palpable lymphadenopathy and had no pancreatic, renal or thyroid dysfunction nor chest pains or visual complaints.

Repeated ultrasound-guided endoscopic biopsy of the right hilar nodal complex and bronchial lavage failed to yield a definitive diagnosis. The aetiology was subsequently presumed benign after exclusion of mycobacterial and fungal disease and non-progression of the disease both clinically and on repeated imaging over a 2-year follow-up period.

However, he presented with episodes of haemoptysis, which were attributed to post-infective bronchiectasis 3 years later. During the subsequent year, he was admitted to hospital thrice and a CT scan showed a right hilar nodal mass with stenosis of the right upper lobe and lower lobe pulmonary arteries with collapse consolidation of the right middle lobe. Right middle lobectomy was recommended after a multidisciplinary team discussion; however, a right pneumonectomy was performed due to severe adhesions at surgery.

The diagnosis of immunoglobin 4-related disease (IgG4-RD) was finally made on histology. Sections of the mass lesion showed extensive storiform fibrosis with obliterative phlebitis and an abundant lymphoplasmacytic infiltrate. Immunohistochemical staining for IgG4 demonstrated >50 IgG4-positive cells per high-power field, meeting the criteria for histology that was highly suggestive of IgG4-RD (Fig. 1D).

The patient was admitted with right-sided hemiparesis and ipsilateral cranial nerve IX, XI, XII palsies 6 months later. CT and magnetic resonance imaging of the brain did not show any underlying parenchymal abnormality or meningitic inflammation, but cerebrospinal fluid analysis showed elevated levels of immunoglobulins (IgGs). Regrettably, subset analysis was not done on this sample. His symptoms resolved with corticosteroids (prednisone at 1 mg/kg) and azathioprine was subsequently initiated as a steroid-sparing agent.

## Discussion

The IgG4 subclass accounts for <5% of total IgG in healthy persons and is the least abundant of all IgG subclasses. It shares >95% homology with other IgG subclasses, but amino acid differences within the second constant domain result in weak or negligible binding to both complement 1q and Fcγ receptors. IgG4 was previously thought not to play a significant role in immune activation; however, advances over the last decade have improved our understanding of the pathophysiology of IgG4-RD, which results from a complex interplay between Th2 cells and B lymphocytes, resulting in the production of dysfunctional plasma cells and other immunogenetic dysregulation responsible for many clinical manifestations of IgG4-RD.^[1]^

IgG4-RD remains a relatively uncommon disease and the first cases of the disease were published in 2003.^[1]^ It usually occurs in the 5th and 6th decade of life, with a male to female ratio of 3:1, which is different to most known auto-immune illnesses.^[1]^ The most common manifestations of IgG4-RD are type 1 auto-immune pancreatitis and sialoadenitis, but retroperitoneal fibrosis with hydronephrosis and renal failure, aortitis with aortic dissection^[2]^ and CNS sequelae secondary to pachymeningitis may occur,^[3]^ demanding urgent diagnosis and intervention.

Intra-thoracic and pulmonary involvement in IgG4-RD is relatively rare, occurring in 15 - 20% of cases and may include mediastinal structures.^[4]^ Pulmonary findings of IgG4-RD include intra-thoracic lymphadenopathy, pleural and bronchial wall thickening, pleural effusion (usually exudative), intra-parenchymal nodules and mass lesions (pseudo-tumours), ground-glass opacification, and in some cases consolidation and fibrosis (Table 1). Fibrosis mimics nonspecific interstitial pneumonia.^[4]^ PFT findings are heterogenous among patients, with some patients (36%) demonstrating no abnormality while 30% had restrictive findings and 34% had obstructive findings.^[5]^

A definitive diagnosis of IgG4-RD requires tissue biopsy and histology usually displays three key features: lymphoplasmacytic infiltration; obliterative phlebitis and storiform fibrosis – the latter being the most important. Mild to moderate eosinophilia may also occur.

Elevated serum levels of IgG4 >1.35 g/L and abundance of IgG4-positive plasma cells in the tissue supports the diagnosis; however, this may also be seen in other diseases such as lymphomas and medium-vessel vasculitides, namely granulomatosis with polyangitis (GPA).^[1,4]^ Isolated elevation of serum IgG4 levels during primary sclerosing cholangitis, inflammatory bowel disease and Hashimoto’s thyroiditis is of uncertain significance, but these disorders do not appear to be part of the spectrum of IgG4-RD. A third of patients with IgG4-RD have normal serum IgG4 levels.^[1]^

Differential diagnosis of the pulmonary manifestations of IgG4-RD includes multicentric Castleman’s disease (MCD), lung carcinoma, other granulomatous diseases (tuberculosis, non-tuberculosis meningitis and sarcoidosis) and ANCA-related mediumvessel vasculitides (eosinophilia with granulomatosis and polyangiitis, and granulomatosis with polyangiitis ). Distinguishing these disease entities both clinically and radiologically can be difficult; however, certain features can assist. MCD demonstrates no active fibrosis, with lower levels of eosinophilic infiltrates in the lung tissue, while IgG4-RD does not usually have positive serology for ANCA antibodies. Sarcoidosis and tuberculosis frequently demonstrate increased serum angiotensin-converting enzyme levels and the presence of granulomas on histological examination of samples, which are not present in IgG4-RD.

Although IgG4-RD may be a risk factor for lung cancer, it is possible for both to be present concomitantly. The presence of malignant cells will exclude IgG4-RD if none of the other histological features are present.^[4]^ Many patients with IgG4-RD have allergic features such as atopy, eczema, asthma and modest peripheral blood eosinophilia, found in up to 40% of patients with IgG4-RD. Thus, atopy does not exclude the diagnosis of IgG4-RD.

The clinical course of IgG4-RD is frequently indolent and symptoms are related to the underlying organ involvement. Often, the diagnosis is made incidentally on imaging and serum levels of C-reactive protein and erythrocytes sedimentation test are seldom elevated. Spontaneous resolution of disease had been reported in a minority of cases.^[1]^ A study of 37 patients with pulmonary disease reported five distinct entities of disease based on radiological findings: bronchovascular; solid nodular; round ground-glass opacity (GGO); alveolar interstitial and alveolar consolidative types. Bronchovascular-type is characterised by a thickening of bronchovascular bundle and interlobular septa, solid nodular-type is characterised by a lung nodule or mass, round GGO type has multiple round-shaped GGO lesions, alveolar interstitial-type is characterised by reticulation, diffuse GGO and honeycombing, and alveolar consolidative-type is distinguished by airspace filling opacities obscuring vasculature in a segmental or lobar distribution. The patients (mean age of 56 years) were followed up for 38 months and outcomes were heterogenous for the different entities, with alveolar consolidative-type and alveolar interstitial-type showing the highest and lowest response rates, respectively.

There is no standardised treatment regimen for IgG4-RD, but most regimens consist of glucocorticoids, usually prednisone (0.6 - 1 mg/kg), which is tapered according to response.^[1]^ Steroid-sparing regimenss consisting of azathioprine (initiating at 50 mg daily) and in cases of relapse, a combination of azathioprine/ glucocorticoids and rituximab have been used.^[5]^

Complications of IgG4-RD depend on the degree, location and progression of fibrosis. The most serious complications are related to the specific organ involved, e.g. aortitis with aortic dissection, pachymeningitis with residual neurological deficit and retroperitoneal fibrosis with hydronephrosis.^[6]^ IgG4-related lung disease usually has a favourable prognosis and severe long-term complications are less frequent.^[5]^

IgG4-RD is a relatively well-described disease entity in developed countries, but there is little information on this rare disease emanating from the African continent. With many other possible disease mimickers, IgG4-RD is an important consideration in patients where the usual diagnosis remains inconclusive.

## Conclusion

This present case highlights an unusual entity of IgG4-RD of the lung and difficulties associated with making a diagnosis. Although initially diagnosed as lung cancer, but later identified as a benign disease process, it took several years and disease progression to facilitate histological diagnosis of IgG4-RD. Furthermore, raised IgG4 levels in the blood or tissue are not sufficient to make a diagnosis, as they may be raised in other conditions. The potential for multisystem involvement is emphasised by the subsequent development of neurological involvement in our patient.

## Figures and Tables

**Table 1:** Disease manifestations of IgG4-RD

**Organ system**	**Disease manifestation**
**Most common**	
Gastro-intestinal tract	Type 1 auto-immune pancreatitis
Head and neck	Mikulicz disease (abnormal enlargement of glandular tissue of the head and neck)
	Sialoadenitis
	Dacryoadenitis
	Orbital pseudotumour
Kidneys	Tubulo-interstitial nephritis
**Less common**	
Lung involvement	Solitary pulmonary nodule
	Ground-glass opacification
	Hilar lymphadenopathy
	Pleural thickening
	Pleural effusion
Mediastinum	Mediastinal fibrosis
	Aortitis
Intracranial	Pachymeningitis
	Hypophysitis
Intra-abdominal	Retroperitonial fibrosis
	Sclerosing cholangitis
**Important differential diagnoses** **to consider (organ involvement-dependent)**	
Lymphoma	
Multicentric Castleman’s disease	
Lung cancer	
Granulomatosis with polyangiitis	
Sarcoidosis	
Pancreatic cancer	
